# Self-reported interpersonal and educational/vocational difficulties in young adults with a history of transient psychotic experiences: findings from a population-based study

**DOI:** 10.1186/s12888-020-03022-z

**Published:** 2021-01-11

**Authors:** Helen Coughlan, Erin Walton-Ball, Eleanor Carey, Colm Healy, Grace O’Regan-Murphy, Aoife Nic Uidhir, Mary C. Clarke, Mary Cannon

**Affiliations:** 1 Department of Psychiatry, Royal College of Surgeons in Ireland, RCSI Education and Research Centre, Beaumont Hospital, Dublin, Ireland; 2 Department of Psychology, Royal College of Surgeons in Ireland, Dublin, Ireland; 3 Department of Psychiatry, Beaumont Hospital Dublin, Dublin, Ireland

**Keywords:** Transient psychotic experiences, Interpersonal difficulties, Educational and vocational difficulties, Functioning

## Abstract

**Background:**

Psychotic experiences (PEs) are not uncommon in young people and are associated with both psychopathology and compromised global functioning. Although psychotic experiences are transient (short-lived, self-resolving and non-recurring) for most people who report them, few studies have examined the association between early transient PEs and later functioning in population samples. Additionally, studies using self-report measures of interpersonal and educational/ vocational difficulties are lacking. The aim of this study was to examine the relationship between transient psychotic experiences and self-reported interpersonal and educational/vocational difficulties in adolescence and young adulthood.

**Methods:**

Participants were 103 young people from a longitudinal population-based study cohort of mental health in Ireland. They attended for baseline clinical interviews in childhood (age 11–13) and were followed up in young adulthood (age 19–25). Participants who reported psychotic experiences at baseline but not at follow-up were classified as having transient psychotic experiences. Data from both time-points were used to examine the association between transient psychotic experiences and self-reported interpersonal and educational/ vocational difficulties in young adulthood using poisson regression modelling.

**Results:**

Young people with a history of transient psychotic experiences reported significantly higher interpersonal (adj IRR: 1.83, 95%ileCI: 1.10–3.02, *p* = .02) and educational/vocational (adj IRR: 2.28, 95%ileCI: 1.43–3.64, *p* = .001) difficulties during adolescence. However, no significant differences in interpersonal (adj IRR: 0.49, 95%ileCI: 0.10–2.30, *p* = .37) or educational/vocational (adj IRR: 0.88, 95%ileCI: 0.37–2.08, *p* = .77) difficulties were found in young adulthood. Self-reported interpersonal and educational/vocational difficulties in young people both with and without a history of transient psychotic experiences decreased between adolescence and young adulthood.

**Conclusions:**

Young people with transient psychotic experiences have increased interpersonal and educational/vocational difficulties in adolescence but these may not persist into the young adult years. This finding indicates that early psychotic experiences may not confer high risk for long-term interpersonal or educational/vocational deficits among young people who experience these phenomena transiently.

## Background

Hallucinations and delusions that occur in the absence of psychotic disorders are commonly referred to as psychotic-like experiences (PEs). PEs are not uncommon, particularly in childhood and adolescence [1], with approximately 17% of 9–12-year-olds reporting these phenomena [2]. By adulthood, the rate declines to an estimated 6% [3]. Notwithstanding this decline, young people who experience PEs have been found to be at higher risk of both concurrent [4, 5] and later psychopathology [1, 6–8] and of multi-morbidity [9, 10]. They have also been found to have higher rates of exposure to a range of childhood adversities, including abuse, victimization and bullying [11–14]. Evidence of an association between childhood and adolescent PEs and global functioning deficits has also been established. Using interviewer-rated measures of global functioning, two previous studies from our research group found that young people who reported childhood PEs were at higher risk of functioning deficits up the age of 21 years [15, 16]. In early adolescence, the association was partly mediated by exposure to childhood adversity [16].

For about 80% of people, PEs are transient experiences that do not reoccur or persist over time [17–19]. This raises questions about long-term functioning outcomes for young people who only experience PEs transiently (i.e. PEs that are short-lived, self-resolving and non-recurring). Evidence on adult outcomes in young people with a history of transient PEs is, however, limited. Most studies are either cross-sectional or use lifetime PEs as their exposure. Few discriminate between transient and reoccurring PEs. Only a small number of longitudinal studies have examined the relationship between transient PEs and later functioning in general population youth samples. Using the Global Assessment Scale (GAS) of the Structured Interview for Prodromal Syndromes (SIPS), Calkins et al (2017) examined functioning among a sample of 8–21 year-olds with and without a history of PEs. Young people who reported PEs at baseline were found to have lower levels of global functioning at follow-up (approximately 2 years later), regardless of whether or not their PEs persisted over time [20]. Using the Global Assessment of Functioning Scale (GAF), a previous study by our research group also found longitudinal evidence of poorer global functioning in young people with a history of childhood PEs during their mid- and late-adolescent years [15]. Compared with young people without any history of PEs, the functioning differences found were evident in young people who reported both transient and recurrent PEs. Findings from these two studies therefore suggest that transient PEs may be a risk factor for later functioning deficits.

Although there is evidence of long-term interpersonal and educational/vocational difficulties among young people who experience PEs few studies have reported on individuals with transient PEs. Furthermore, studies that have examined transient PEs and later functioning have relied on global, interviewer-rated measures of functioning, for which clinical symptoms have been found to be stronger predictors of functioning scores than individuals’ social and occupational functioning [21]. Although self-reported measures psychosocial functioning have been established as reliable and valid [22], few studies have reported on self-report measures of functioning difficulties in young people with a history of PEs. In this context, the aim of the current study was to examine the association between transient PEs and self-reported interpersonal and educational/vocational difficulties. Based on evidence to date, we hypothesised that young adults with a history of childhood PEs that did not reoccur or persist into young adulthood (i.e. transient PEs) would report higher levels of interpersonal and educational/ vocational difficulties throughout adolescence and into young adulthood than young people who had never reported PEs.

## Methods

### Participants

Participants were recruited from primary schools in Dublin and environs, Ireland, as part of the Adolescent Brain Development (ABD) study. Recruitment details for this study have previously been reported [23]. Briefly, 1131 pupils aged 11–13 years consented to take part in a survey of childhood psychopathology, of whom 211 participants completed further in-depth clinical interviews and neurocognitive assessment. All 211 interview participants were invited to attend for follow-up clinical interviewing and neurocognitive testing approximately 10 years later. Of the baseline sample, 103 participants returned. These individuals made up the sample for the current study. The age range of the young adult follow-up sample was 19–25 years.

Ethical approval for the study was granted by the Beaumont Hospital Dublin Research Ethics Committee.

### Design and measurement

The current study reported three types of variable that were grouped as follows: 1. Exposure measure: whether or not participants had experienced transient PEs, 2: Outcome measures: indicators of long-term functioning in participants. These were defined as self-reported interpersonal and educational/vocational difficulties. 3: Potential confounders: variables that could be associated with both the exposure and/or the outcome. These were gender, socioeconomic status, baseline psychopathology, childhood adversity, childhood peer problems and childhood educational problems. Criteria for each of the variables is defined below.

### Exposure measure

#### Psychotic experiences

Psychotic experiences (PEs) were assessed at both baseline and follow-up using the PE assessment protocol designed for the ABD study [23]. At both time-points, as part of the psychopathology assessment, all participants were asked about any lifetime experiences of hallucinatory or delusional experiences. For any experiences reported, detailed data about the phenomenology, attribution, impact and level of distress were recorded by study interviewers. At both baseline and follow-up, these data were firstly independently reviewed by three members of the study team, two of whom were qualified psychiatrists. Following the independent rating process, a consensus meeting was held involving all three raters to discuss and finalise PE ratings. At baseline, rates of PEs were based on lifetime experiences of PEs (no participant met criteria for a psychotic disorder). At follow-up, they were based on experiences of PEs over the previous 12-months only.

For the purposes of the current study, participants were classified as having Transient PEs if they met PE criteria at baseline but not at follow-up. Participants who did not meet PE criteria at either baseline or follow-up were classified as controls. Participants who reported PEs at both time-points were classified as having Reoccurring PEs and those who reported PEs at follow-up but not at baseline were classified as having New Onset PEs; the latter two groups of participants were not included in analysis.

### Outcome measure

#### Self-reported adolescent and young adult interpersonal and educational/vocational difficulties

Self-reported interpersonal and educational/vocational difficulties were determined using data from an adapted version of the Stressful Life Events Schedule, Version 3.01. The SLES is a 79-item self-report questionnaire that asks about a range of stressful life experiences and difficulties [24]. The SLES was reviewed to identify questions relating to both interpersonal and educational/vocational difficulties. These areas of interest were chosen to reflect core aspects of externally-rated measures of functioning, such as the Global Assessment of Functioning. Seven questions were identified that related to interpersonal difficulties (e.g. Have you had an increase in arguments and/or relationship problems with any close friends?). Another seven were identified that related to educational/vocational difficulties (e.g. Have you failed or done poorly on any major exams or standardised tests?). For both, each of the 7 self-report responses was coded using binary (Yes/No) coding to indicate the presence or absence of the experience. Participants were also asked to indicate the age(s) at which they had any experience they endorsed. This was used to categorise reported difficulties into the time periods of adolescence (if reported as occurring between the ages 13–18 inclusive) and young adulthood (if reported as occurring at age 19 years+). Self-reported difficulties during these two periods were then summed to create an interpersonal (0–7) and an educational/vocational (0–7) score for each participant during adolescence and young adulthood. Interpersonal and educational difficulties at both time points were both treated as count variables.

### Potential confounders

#### Demographic characteristics

At baseline (aged 11–13 years), data were gathered on participants’ gender, date of birth and socioeconomic status (SES). Parental occupation was used as a proxy measure for SES. Participants were classified into one of seven SES groups based on a 7-point Irish Social Class Scale from the Irish Central Statistics Office. Participants with parents who reported professional and managerial roles were assigned values of 1 and 2 respectively and those with parents who reported working in non-manual skilled, skilled manual, semiskilled manual, unskilled manual labour or were unemployed, were assigned to the values 3, 4, 5, 6 and 7 respectively.

#### Psychopathology

At baseline, both the participant and his/her parent or guardian were interviewed. Psychopathology was measured using the Schedule for Affective Disorders and Schizophrenia in School-aged Children, Present and Lifetime Version (K-SADS-PL) [25]; this is a clinical interview schedule used to assess for current (past 4 weeks) and lifetime Axis I DSM-IV [26] mental disorders. At follow-up, psychopathology was assessed using the semi-structured Structured Clinical Interview for DSM-V, Research Version (SCID-5-RV) [27]. The SCID-5 was used to determine current (past 4 weeks) and lifetime DSM-V mental disorders. Psychopathology was defined as meeting criteria for Axis-1 DSM-IV diagnosis during clinical assessments at each time point.

#### Childhood adversity

As part of the K-SADS-PL, all participants and their parents/guardians were systematically asked about any history of physical or sexual abuse or being the victim of bullying, all of which have an established association with PEs [11]. All participants rated their level of distress in response to being bullied on a scale from 0 to 10 (0=no distress). The median distress rating in those who were bullied was 4/10 and this was used as a cut-off score to classify bullying as an adverse exposure within the sample. For the current study, childhood adversity was treated as a dichotomous variable (0=no childhood adversity reported, 1=any report of physical and/or sexual abuse and/or bullying rated 4/10 or above). This measure was recorded in order to control for any confounding effects of childhood adversity on the relationship between PE and long-term functioning.

#### Childhood educational and interpersonal difficulties

Two proxy measures were used to examine childhood interpersonal and educational difficulties reported at baseline. Childhood educational difficulties were examined using data on whether the child required special educational supports (yes/no) and thus was recorded as a dichotomous variable. Childhood interpersonal difficulties were examined using data from the the Strengths and Difficulties Questionnaire (SDQ) [28], which all participating children completed as part of the baseline study. The SDQ is a well-validated brief survey instrument that assesses for psychological attributes in young people. The instrument is comprised of 5 sub-scales, one of which screens for peer relationship problems. Results can be analysed as both continuous scores and/or as categorical scores indicating normal, borderline and abnormal levels of difficulties for each subscale. For the purposes of the current study and in line with scoring ranges established by Goodman et al [28], peer relationship problem scores were dichotomised into either normal (score of 0–3) or borderline-abnormal (score of 4–10).

### Analysis

All analyses were conducted using Stata version 15. An α of 0.05 was used to classify *p*-values as significant or non-significant in all statistical comparisons.

#### Attrition analysis

Chi-square and independent samples t-tests were used to test for differences in gender, age, PEs, the need for special educational supports, peer problems in childhood, childhood adversity and Axis 1 DSM-IV disorders at baseline between those ABD cohort participants who did (*N*=103) and did not (*N*=108) return for follow-up.

#### Demographic analysis

Chi-square and independent samples t-tests were also used to test for group differences in gender, SES, DSM-IV mental disorders at baseline, childhood adversity, childhood peer and interpersonal problems and the mean age at each time-point of the Transient PEs and control groups. Differences in the number of interpersonal and educational/vocational difficulties between males and females in adolescence and young adulthood were examined using non-parametric t-tests.

#### Group differences in self-reported interpersonal and educational/vocational difficulties

At both time-points, interpersonal and educational/vocational difficulties were treated as count variables. There was a right skew in both variables indicating a notable proportion of zero endorsement. For this reason, the panel outcome data were analysed using xtpoisson command, a repeated measures analysis specific to count variables with a poisson distribution. We report incident rate ratio (IRR) for group (PEs versus control) and time (adolescence versus young adulthood). We report the overall interaction between group and time as well as the time-specific contrast between the groups (PEs versus controls in adolescence and young adulthood separately). Interpersonal and educational/vocational difficulties were analysed separately as outcome variables. For both outcomes, we report three models with different levels of adjustment for potential confounding. Model 1 was adjusted for gender and SES. Model 2 was adjusted for gender, SES, childhood psychopathology and childhood adversity. Finally, model 3 was adjusted for gender, SES, childhood psychopathology, childhood adversity, childhood peer problems and childhood educational problems.

## Results

### Attrition comparison

Comparison of baseline data between those who did and those who did not return for follow-up revealed no significant differences in gender (χ^2^(1) = 0.01, *p* = .94), age (t=1.26, *p*=.21), childhood PEs (χ^2^(1) = 2.66, *p* = .10), SES (t=− 1.64, *p*=.10), need for special educational support (χ^2^(1) = 2.60, *p* = .11), SDQ borderline/abnormal peer problems (χ^2^(1) =0.24, *p* = .63), childhood adversity (χ^2^(1) =2.16, *p* = .14) or childhood psychopathology (χ^2^(1) = 0.44, *p* = .51).

### Sample characteristics

Young adults who attended at follow-up (*N*=103; 46.9% male, 53.1% female) were aged between 19 and 25 years. Of those, 65.0% (*N*=67) did not report PEs at either baseline or follow-up. These individuals made up the control group for this study. A further 28.2% (*N*=29) reported PEs at baseline but not at follow-up. These individuals made up the Transient PE group for the current study (referred to hereafter as the PE group). The remaining 6.8% (*N*=7) of individuals reported either new onset PEs at follow-up (*N*=5) or had recurrent PEs (i.e. met criteria at both baseline and follow-up) (*N*=2). Due to the small numbers of those with either new onset or recurrent PEs, data on these seven individuals were not examined in this study.

No significant differences were found between the PE and control groups in relation to age, SES status, baseline psychopathology, educational or peer problems in childhood. There was, however, a non-significant trend for childhood psychopathology (*p*=0.06), with those in the PE group found to have higher rates of childhood psychopathology. Significant gender differences were found between groups. Those with PEs had significantly higher rates of childhood adversity (see Table 1).

**Table 1:** Sample characteristics

	Transient PE Group (*N*=29)	Control Group (N=67)	***P*** value
Gender			**0.016**
Male	19 (65.5%)	26 (38.8%)	
Female	10 (34.5%)	41 (61.2%)	
Mean age at baseline	11.66	11.72	0.682
Mean age at follow-up	20.72	21.09	0.221
Mean SES^a^	2.64	2.23	0.178
Met criteria for lifetime DSM-IV mental disorder at baseline	14 (48.3%)	19 (28.4%)	0.059
Met criteria for current DSM-V mental disorder at follow-up	8 (27.6%)	4 (6.0%)	**0.003**
Experienced childhood adversity^b^	23 (79.3%)	33 (49.3%)	**0.006**
Borderline or abnormal score on SDQ peer problems subscale at baseline	4 (13.8%)	6 (9.0%)	0.486
Required special educational services at baseline	10 (34.5%)	13 (19.4%)	0.111

^a^ *N* = 93 due to missing SES data

^b^ Defined as any exposure to childhood sexual abuse physical abuse or bullying as reported at baseline

Significant outcomes in bold

### Relationship between transient PEs and self-reported adolescent and young adult interpersonal difficulties

In adolescence, the median number of interpersonal difficulties reported was 1 (range 0–6). There was no significant difference between male and female participants in the number of interpersonal difficulties reported in adolescence (z = 0.35, *p* =.73). In young adulthood most participants did not report interpersonal difficulties (median = 0, range 0–2). There was no significant difference between male and female participants in the number of interpersonal difficulties reported in young adulthood (z = − 0.09, *p* =.93).

Our main analysis indicated that, when controlling for gender and SES, there was a significant effect of group, indicating that participants with Transient PEs had significantly more interpersonal difficulties than controls (IRR: 1.79, 95%ileCI: 1.06–3.01, *p* = .027). There was also a significant main effect of time, indicating a reduction in the number of difficulties reported in young adulthood relative to adolescence (IRR: 0.18, 95%ileCI: 0.09–0.35, *p* < .001). Additionally, there was, a significant interaction between group and time (Chi^2^ = 6.39, *p* = 0.04). This interaction indicated that participants with Transient PEs reported more interpersonal difficulties than controls in adolescence (IRR: 1.79, 95%ileCI: 1.07–3.01, *p* = .03) but not in young adulthood (IRR: 0.48, 95%ileCI: 0.10–2.27, *p* = .36). The predicted number of events for each group in adolescence and young adulthood (based on the estimated marginal means) are displayed in Table 2.

**Table 2:** Estimated marginal mean self-reported interpersonal difficulties scores in adolescence and adulthood in Transient PEs and control groups

Measure	Group	Model 1 (SE)^a^	Model 2 (SE) ^b^	Model 3 (SE) ^c^
Adolescence	Transient PEs	1.52 (0.31)*	1.57 (0.33)*	1.55 (0.30)*
	Control	0.85 (0.13)*	0.83 (0.13)*	0.85 (0.13)*
Young Adulthood	Transient PEs	0.07 (0.05)	0.07 (0.06)	0.08 (0.05)
	Control	0.15 (0.05)	0.15 (0.05)	0.15 (0.05)

*N* = 94 due to missing SLES data for one participant

^a^Adjusted for gender and SES; ^b^Adjusted for gender, SES, baseline psychopathology and childhood adversity; ^c^Adjusted for gender, SES, baseline psychopathology, childhood adversity, childhood peer problems and childhood educational problems

*p< 0.05; **p< 0.01; ***p< 0.001

#### Model 2

Additional adjustment for baseline psychopathology and childhood adversity did not alter the interpretation of findings from the main effects analysis (Group: IRR: 1.89, 95%ileCI: 1.11–3.21, *p* = .019; Time: IRR: 0.18, 95%ileCI: 0.09–0.36, *p* < .001), interaction (Chi^2^ = 6.99, *p* = 0.03) or simple effects analysis (adolescence: IRR: 1.88, 95%ileCI: 1.11–3.21, *p* = .02; young adulthood: IRR: 0.51, 95%ileCI: 0.11–2.40, *p* = .39).

#### Model 3

Further adjustment for childhood peer problems and childhood educational problems again did not alter our interpretation of the findings from the main effect analysis (Group: IRR: 1.83, 95%ileCI: 1.10–3.02, *p* = .019; Time: IRR: 0.18, 95%ileCI: 0.09–0.36, *p*< .001), interaction (Chi^2^ = 6.89, *p*= 0.03) or simple effects analysis (IRR: 1.83, 95%ileCI: 1.10–3.02, *p* = .02; young adulthood: IRR: 0.49, 95%ileCI: 0.10–2.30, *p* = .37).

### Relationship between transient PEs and self-reported adolescent and young adult educational/vocational difficulties

In adolescence and young adulthood, most participants did not report educational/vocational difficulties (adolescent median: 0, range 0–5; young adulthood median: 0, range 0–2). Male participants reported significantly more educational/vocational difficulties than female participants did in adolescence (z = 4.04, *p* <.001) but not in young adulthood (z = 1.55, *p* = .122).

Our main analysis indicated that, when controlling for gender and SES, there was a significant effect of Group indicating that participants with Transient PEs had significant more educational/vocational difficulties than controls (IRR: 1.99, 95%ileCI: 1.26–3.14, *p* = .003). There was also a significant main effect of time indicating a reduction in the number of difficulties reported in young adulthood relative to adolescence (IRR: 0.43, 95%ileCI: 0.24–0.78, *p* = .005). Additionally, there was, a significant interaction between group and time (Chi^2^ = 8.84 *p* = 0.01). This interaction indicated that participants with Transient PEs reported more educational/vocational difficulties than controls in adolescence (IRR: 1.98, 95%ileCI: 1.25–3.14, *p* = .003) but not in young adulthood (IRR: 0.87, 95%ileCI: 0.37–2.06, *p* = .76). The predicted number of events for each group in adolescence and young adulthood (based on the estimated marginal means) are displayed in Table 3.

**Table 3:** Estimated marginal mean self-reported educational/vocational difficulties scores in adolescence and adulthood in Transient PEs and control groups

Measure	Group	Model 1 (SE)^a^	Model 2 (SE) ^b^	Model 3 (SE) ^c^
Adolescence	Transient PEs	1.26 (0.20)***	1.39 (0.22)***	1.38 (0.22)***
	Control	0.63 (0.10)***	0.59 (0.10)***	0.60 (0.10)***
Young Adulthood	Transient PEs	0.24 (0.09)	0.25 (0.09)	0.24 (0.09)
	Control	0.27 (0.07)	0.27 (0.07)	0.28 (0.07)

*N* = 94 due to missing SLES data for one participant

*p< 0.05; **p< 0.01; ***p< 0.001

^a^Adjusted for gender and SES; ^b^Adjusted for gender, SES, baseline psychopathology and childhood adversity; ^c^Adjusted for gender, SES, baseline psychopathology, childhood adversity, childhood peer problems and childhood educational problems

#### Model 2

Additional adjustment for baseline psychopathology and childhood adversity did not alter the interpretation of the findings from the main effects analysis (Group: IRR: 2.35, 95%ileCI: 1.47–3.75, *p* < .001; Time: IRR: 0.46, 95%ileCI: 0.26–0.82, *p* = .008), interaction (Chi^2^ = 13.15, *p* = 0.001) or simple effects analysis (adolescence: IRR: 2.35, 95%ileCI: 1.48–3.75, p < .001; young adulthood: IRR: 0.91, 95%ileCI: 0.39–2.14, *p* = .83).

#### Model 3

Further adjustment for childhood peer problems and childhood educational problems again did not alter our interpretation of the findings from the main effect analysis (Group: IRR: 2.28, 95%ileCI: 1.43–3.64, *p* < .001; Time: IRR: 0.46, 95%ileCI: 0.26–0.82, *p* = .008), interaction (Chi^2^ = 12.19, *p*=.002) or simple effects analysis (adolescence: IRR: 2.28, 95%ileCI: 1.43–3.64, *p* = .001; young adulthood: IRR: 0.88, 95%ileCI: 0.37–2.08, *p* = .77).

**Fig. 1:**
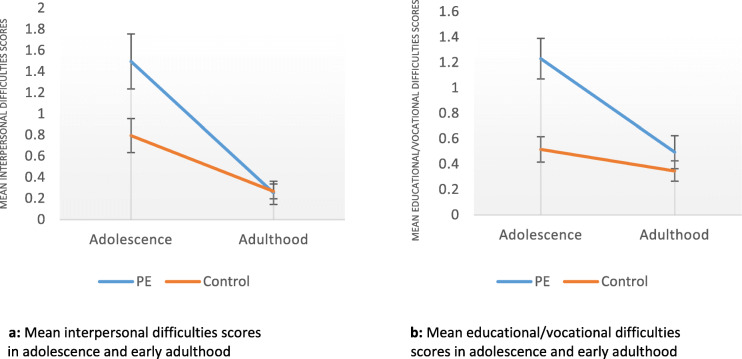
Mean interpersonal and educational/vocational difficulties scores in adolescence and young adulthood in the Transient psychotic experiences and control groups*. *Adjusted for gender, SES, baseline psychopathology, childhood adversity, childhood peer problems and childhood educational problems. Bars indicate standard error

As reflected in Fig. 1, the between group convergence in estimated marginal mean self-reported interpersonal (Fig. 1a) and educational/vocational difficulties (Fig. 1b) scores in young adulthood could not be explained by worsening educational/vocational and interpersonal difficulties in the control group, as difficulties were observed to decrease in both groups over time.

## Discussion

Findings from this study only partly supported our hypothesis that transient childhood PEs would be associated with self-reported interpersonal and educational/vocational difficulties throughout adolescence and into young adulthood. Specifically, we found that young adults with a history of Transient PEs reported higher levels of interpersonal and educational/vocational difficulties during their adolescent years. However, these differences were no longer present in young adulthood. Moreover, they could not be explained by increasing levels of difficulties in our control group as both interpersonal and educational/vocational difficulties reduced in all groups over time.

The finding of higher levels of educational/vocational difficulties and problematic interpersonal relationships in adolescence in our PE group was consistent with existing evidence from interviewer-rated functioning deficits in young people with a history of transient PEs [15, 20]. As found by previous studies, the differences between groups during adolescence could not be fully explained by childhood psychopathology or by childhood adversity [16]. However, our young adult findings contrasted with evidence from other studies using interviewer-rated measures, which have identified functional deficits up to the age of 21 among young people with a history of transient PEs [15, 20]. One potential explanation for this difference is that educational/vocational and interpersonal difficulties associated with transient PEs may themselves have been transient. Furthermore, the post-adolescent period may represent a critical time for improvements in difficulties in young people with a history of transient PEs. There is some support for this hypothesis from limited evidence regarding stress reactivity. In their study, Cullen et al. found that children with PEs had both high levels of stress exposure and heightened distress responses [30]. However, in their twin study, Collip and colleagues found that adults with transient PEs had lower levels of stress reactivity than those with persistent PEs [31]. This suggests that transient PEs may not be a strong indicator of risk for future difficulties and sensitivity to stress in adulthood.

The results of this study also complement findings from the field of resilience research, in that positive environmental, relational and personal factors can have a remediation effect on the mental health and functioning of children and adolescents [32]. The Kauai Longitudinal Study found that, by mid-life the majority of high risk adolescents, including those with poor mental health, poor coping and delinquency, were functioning well [33, 34]. Furthermore, they were satisfied with their interpersonal relationships and had been contributing to their communities. Thus, the improvements observed in this study by young adulthood could, at least in part, be explained by the potential for vulnerable youth to catch up with their less vulnerable peers during the early to mid-adult years. There is some support for this hypothesis based on findings from a qualitative study that was undertaken as part of the Adolescent Brain Development (ABD) study [35]. The study involved in-depth qualitative interviews with 17 participants (aged 18–21 years), all of whom had reported experiencing early PEs. Findings from that study found that a number of young people with a history of PEs reported high levels of life satisfaction, well-being, educational functioning and positive interpersonal relationships, despite early exposure to childhood adversity. What differentiated young people with positive outcomes from those who were struggling was the presence of a supportive adult attachment figure and opportunities to contribute to society. Both of the aforementioned factors have been found to be protective against poor psychosocial outcomes in life and to nurture resilience [34, 36, 37]. The key role that attachment relationships and other protective factors have in supporting positive outcomes in young adulthood in young people with a history of PEs may therefore be relevant in understanding findings in this study. Attachment, for example, has been found to mediate the relationship between early adversity and PEs [38–40] and between adversity and psychological distress/wellbeing [41]. Similarly, positive parent-child relationships during adolescence have recently been identified as mediators of the relationship between early adversity and PEs [29], and between PEs and other psychopathology [42] in youth populations. Longitudinal research on the role of psychosocial factors in the relationship between PEs and functioning could provide valuable insight into potential mediators of poor long-term functioning outcomes in individuals with experiences of early adversity, PE and psychopathology.

One alternative explanation for our findings is that they reflect a positive self-report bias among participants in this study. Thus, that young adults may have had more positive subjective perceptions of their relationships and their educational or vocational experiences than those appraised externally. However, this was not fully supported by our finding of significant differences reported in adolescence. Alternatively, it may reflect evidence that global functioning scales may lack sensitivity in measuring or integrating psychosocial functioning [21, 43]. Our self-reported findings on interpersonal and educational/vocational difficulties may have therefore provided a more accurate indication of difficulties in these specific functioning domains. This is supported by evidence that self-reported measures of psychosocial functioning have been found to be both reliable and valid [22]. Further research using validated self-report measures of functioning would, however, be useful.

Additionally, although there has been growing evidence that early PEs may be a marker of functional deficits that cannot be fully explained by psychopathology, evidence has also suggested that the relationship between PEs and functioning may be affected by multiple additional factors that interact dynamically and synergistically. Young people with experiences of early adolescent PEs have also been found to be at increased risk for a range of later neurocognitive [44], interpersonal [45], attachment [35] and psychopathological [17, 18] difficulties. Each of these could also have effects on social, educational, vocational and interpersonal functioning. A combination of these factors is likely to have increased that risk further. Young people with PEs are also more likely to have had a history of childhood adversity and trauma [11–14], which are known to affect brain development [46], social functioning [47, 48], cognitive functioning [49, 50] and mental health generally [51]. Conversely, factors such as lower levels of adversity, higher self-esteem and spirituality have been found to be protective against poor outcomes, even among people who have persistent PEs [52]. The sample size of the current study precluded the examination of these potential mediators or moderators.

This study included full clinical interviews, consensus meetings to assess for PEs and a community-based sampling approach, which eliminated the risk for bias resulting from health-care seeking behaviour or snowball sampling. Limitations of the study include the small sample size, which increased the potential for type II error. The small sample size also precluded us from comparing transient with reoccurring or new onset PEs, which would have enhanced the study considerably. Furthermore, our classification of control and Transient PEs subgroups was based on data taken at two time points, approximately 10 years apart. Therefore, they have not accounted for any experiences of PEs that may have occurred in the interim time period. Additionally, we note that the SLES enquired about life events and functioning both currently and retrospectively, which potentially may have decreased the accuracy of some of the responses.

## Conclusions

This study provides new and encouraging evidence demonstrating that, while young people with a history of early but transient PEs reported higher levels of interpersonal and educational/vocational difficulties during their adolescent years, these difficulties may not persist into young adult years. In light of this, we tentatively propose that, on their own, transient PEs may not be a potent risk factor for longitudinal interpersonal and educational/vocational difficulties into the adult years. In the context of the known protective effects of secure attachment relationships, positive peer relationships, educational connection and achievement, trauma-, attachment- and resilience-focused interventions may therefore help to maximise positive outcomes for young people who report transient PEs.

## Data Availability

The datasets generated and/or analysed during the current study are not publicly available but full details on the study methods, measures, protocols and data can be made available by the corresponding author on reasonable request.

## Abbreviations

GAF: Global Assessment of Functioning
GAS: Global Assessment Scale
K-SADS-PL: Schedule for Affective Disorders and Schizophrenia in School-aged Children, Present and Lifetime Version
PEs: Psychotic Experiences
PE group: For this study, refers to individuals classified as having transient psychotic experiences, defined as having reported psychotic experiences at baseline but not at young adult follow-up
SES: Socioeconomic Status
SCID: Structured Clinical Interview for DSM-V
SDQ: The Strength and Difficulties Questionnaire
SIPS: Structured Interview for Prodromal Syndromes

## References

[1] Sullivan S, Kounali D, Cannon M, David A, Fletcher P, Holmans P, et al. A population-based cohort study examining the incidence and impact of psychotic experiences from childhood to adulthood, and prediction of psychotic disorder. Am J Psychiatr. 2019; 10.1176/appi.ajp.2019.19060654. 10.1176/appi.ajp.2019.19060654 31906710

[2] Kelleher I, Connor D, Clarke MC, Devlin N, Harley M, Cannon M (2012). Prevalence of psychotic symptoms in childhood and adolescence: a systematic review and meta-analysis of population-based studies. Psychol Med.

[3] McGrath JJ, Saha S, Al-Hamzawi A, Alonso J, Bromet EJ, Bruffaerts R (2015). Psychotic experiences in the general population: a cross-national analysis based on 31 261 respondents from 18 countries. JAMA psychiatry.

[4] Varghese D, Scott J, Welham J, Bor W, Najman J, O'callaghan M (2009). Psychotic-like experiences in major depression and anxiety disorders: a population-based survey in young adults. Schizophr Bull.

[5] Barragan M, Laurens KR, Navarro JB, Obiols JE (2011). Psychotic-like experiences and depressive symptoms in a community sample of adolescents. Eur Psychiatry.

[6] Fisher HL, Caspi A, Poulton R, Meier MH, Houts R, Harrington H (2013). Specificity of childhood psychotic symptoms for predicting schizophrenia by 38 years of age: a birth cohort study. Psychol Med.

[7] Poulton R, Caspi A, Moffitt TE, Cannon M, Murray R, Harrington H (2000). Children's self-reported psychotic symptoms and adult schizophreniform disorder: a 15-year longitudinal study. Arch Gen Psychiatry.

[8] Healy C, Gordon AA, Coughlan H, Clarke M, Kelleher I, Cannon M (2018). Do childhood psychotic experiences improve the prediction of adolescent psychopathology? A longitudinal population-based study. Early Intervention Psychiatry.

[9] Kelleher I, Keeley H, Corcoran P, Lynch F, Fitzpatrick C, Devlin N (2012). Clinicopathological significance of psychotic experiences in non-psychotic young people: evidence from four population-based studies. Br J Psychiatry.

[10] Kelleher I, Devlin N, Wigman JT, Kehoe A, Murtagh A, Fitzpatrick C (2014). Psychotic experiences in a mental health clinic sample: implications for suicidality, multimorbidity and functioning. Psychol Med.

[11] Coughlan H, Cannon M (2017). Does childhood trauma play a role in the aetiology of psychosis? A review of recent evidence. BJPsych Advances.

[12] Kelleher I, Keeley H, Corcoran P, Ramsay H, Wasserman C, Carli V (2013). Childhood trauma and psychosis in a prospective cohort study: cause, effect, and directionality. Am J Psychiatry.

[13] Arseneault L, Cannon M, Fisher HL, Polanczyk G, Moffitt TE, Caspi A (2011). Childhood trauma and children's emerging psychotic symptoms: a genetically sensitive longitudinal cohort study. Am J Psychiatry.

[14] Kelleher I, Harley M, Lynch F, Arseneault L, Fitzpatrick C, Cannon M (2008). Associations between childhood trauma, bullying and psychotic symptoms among a school-based adolescent sample. Br J Psychiatry.

[15] Healy C, Campbell D, Coughlan H, Clarke M, Kelleher I, Cannon M (2018). Childhood psychotic experiences are associated with poorer global functioning throughout adolescence and into early adulthood. Acta Psychiatr Scand.

[16] Kelleher I, Wigman JT, Harley M, O'Hanlon E, Coughlan H, Rawdon C (2015). Psychotic experiences in the population: association with functioning and mental distress. Schizophr Res.

[17] Linscott RJ, van Os J (2013). An updated and conservative systematic review and meta-analysis of epidemiological evidence on psychotic experiences in children and adults: on the pathway from proneness to persistence to dimensional expression across mental disorders. Psychol Med.

[18] Zammit S, Kounali D, Cannon M, David AS, Gunnell D, Heron J (2013). Psychotic experiences and psychotic disorders at age 18 in relation to psychotic experiences at age 12 in a longitudinal population-based cohort study. Am J Psychiatry.

[19] Van Os J, Linscott RJ, Myin-Germeys I, Delespaul P, Krabbendam L (2009). A systematic review and meta-analysis of the psychosis continuum: evidence for a psychosis proneness–persistence–impairment model of psychotic disorder. Psychol Med.

[20] Calkins ME, Moore TM, Satterthwaite TD, Wolf DH, Turetsky BI, Roalf DR (2017). Persistence of psychosis spectrum symptoms in the Philadelphia neurodevelopmental cohort: a prospective two-year follow-up. World Psychiatry.

[21] Rudolf H, Moos AC (2002). Nichol AB global assessment of functioning ratings and the allocation and outcomes of mental health services. Psychiatr Serv.

[22] Zimmerman M, Ruggero CJ, Chelminski I, Young D, Posternak MA, Friedman M (2006). Developing brief scales for use in clinical practice: the reliability and validity of single-item self-report measures of depression symptom severity, psychosocial impairment due to depression, and quality of life. J Clin Psychiatry.

[23] Kelleher I, Murtagh A, Molloy C, Roddy S, Clarke MC, Harley M (2012). Identification and characterization of prodromal risk syndromes in young adolescents in the community: a population-based clinical interview study. Schizophr Bull.

[24] Williamson DE, Birmaher B, Ryan ND, Shiffrin TP, Lusky JA, Protopapa J (2003). The stressful life events schedule for children and adolescents: development and validation. Psychiatry Res.

[25] Kaufmann J, Birmaher B, Brent D, Rao U, Ryan N (1996). Kiddie-SADS-Present and Lifetime Version (K-SADS-PL).

[26] American Psychiatric Association. Diagnostic and Statistical Manual of Mental Disorders. 5th Edition ed. Washington DC: APA; 2013.

[27] First MB, Williams JBW, Karg RS, Spitzer RL (2015). Structured clinical interview for DSM-5-research version (SCID-5 for DSM-5, research version; SCID-5-RV).

[28] Goodman R, Ford T, Simmons H, Gatward R, Meltzer H (2003). Using the strengths and difficulties questionnaire (SDQ) to screen for child psychiatric disorders in a community sample. Int Rev Psychiatry.

[29] McMahon EM, Corcoran P, Keeley H, Clarke M, Coughlan H, Wasserman D, et al. Risk and protective factors for psychotic experiences in adolescence: a population-based study. Psychol Med. 2020:1–9 10.1017/s0033291719004136. 10.1017/S0033291719004136 32026792

[30] Cullen AE, Fisher HL, Roberts RE, Pariante CM, Laurens KR (2014). Daily stressors and negative life events in children at elevated risk of developing schizophrenia. Br J Psychiatry.

[31] Collip D, Wigman JTW, Myin-Germeys I, Jacobs N, Derom C, Thiery E (2013). From epidemiology to daily life: linking daily life stress reactivity to persistence of psychotic experiences in a longitudinal general population study. PLoS One.

[32] Zolkoski SM, Bullock LM (2012). Resilience in children and youth: a review. Child Youth Serv Rev.

[33] Werner EE (2005). Resilience and recovery: findings from the Kauai longitudinal study. Research Policy Practice Children’s Mental Health.

[34] Werner EE (2004). Journeys from childhood to midlife: risk, resilience, and recovery. Pediatrics..

[35] Coughlan H, Healy C, Sheaghdha ÁN, Murray G, Humphries N, Clarke M, et al. Early risk and protective factors and young adult outcomes in a longitudinal sample of young people with a history of psychotic-like experiences. Early Intervention Psychiatry. 2019; 10.1111/eip.12855. 10.1111/eip.12855 31310453

[36] Bowlby J (1977). The making and breaking of Affectional bonds: I. Aetiology and psychopathology in the light of attachment theory. Br J Psychiatry.

[37] Dooley B, O'Connor C, Fitzgerald A, O'Reilly A (2019). My world survey 2: national study of youth mental health in Ireland.

[38] Berry K, Barrowclough C, Wearden A (2007). A review of the role of adult attachment style in psychosis: unexplored issues and questions for further research. Clin Psychol Rev.

[39] Berry K, Barrowclough C, Wearden A (2008). Attachment theory: a framework for understanding symptoms and interpersonal relationships in psychosis. Behav Res Ther.

[40] Carr SC, Hardy A, Fornells-Ambrojo M (2018). Relationship between attachment style and symptom severity across the psychosis spectrum: a meta-analysis. Clin Psychol Rev.

[41] Corcoran M, McNulty M (2018). Examining the role of attachment in the relationship between childhood adversity, psychological distress and subjective well-being. Child Abuse Negl.

[42] Healy C, Coughlan H, Clarke M, Kelleher I, Cannon M. What mediates the longitudinal relationship between psychotic experiences and psychopathology? J Abnorm Psychol. 2020. https://psycnet.apa.org/doi/10.1037/abn0000523. 10.1037/abn0000523 32309957

[43] Aas IHM (2010). Global assessment of functioning (GAF): properties and frontier of current knowledge. Ann General Psychiatry.

[44] Carey E, Dooley N, Gillan D, Healy C, Coughlan H, Clarke M (2019). Fine motor skill and processing speed deficits in young people with psychotic experiences: a longitudinal study. Schizophr Res.

[45] Healy C, Coughlan H, Williams J, Clarke M, Kelleher I, Cannon M. Changes in self-concept and risk of psychotic experiences in adolescence: a longitudinal population based cohort study. J Child Psychol Psychiatry. 2019; 10.1111/jcpp.13022. 10.1111/jcpp.13022 30771222

[46] Seo D, Tsou KA, Ansell EB, Potenza MN, Sinha R (2014). Cumulative adversity sensitizes neural response to acute stress: association with health symptoms. Neuropsychopharmacology..

[47] Johnson R, Browne K, Hamilton-Giachritsis C (2006). Young children in institutional Care at Risk of harm. Trauma Violence Abuse.

[48] Stain HJ, Brønnick K, Hegelstad WTV, Joa I, Johannessen JO, Langeveld J (2013). Impact of interpersonal trauma on the social functioning of adults with First-episode psychosis. Schizophr Bull.

[49] Lupien SJ, McEwen BS, Gunnar MR, Heim C (2009). Effects of stress throughout the lifespan on the brain, behaviour and cognition. Nat Rev Neurosci.

[50] Aas M, Dazzan P, Mondelli V, Melle I, Murray RM, Pariante CM. A systematic review of cognitive function in first-episode psychosis, including a discussion on childhood trauma, stress and inflammation. Frontiers in Psychiatry. 2014;4 . 10.3389/fpsyt.2013.00182 PMC3884147 24409157

[51] Kessler RC, McLaughlin KA, Green JG, Gruber MJ, Sampson NA, Zaslavsky AM (2010). Childhood adversities and adult psychopathology in the WHO world mental health surveys. Br J Psychiatry.

[52] Peters E, Ward T, Jackson M, Morgan C, Charalambides M, McGuire P (2016). Clinical, socio-demographic and psychological characteristics in individuals with persistent psychotic experiences with and without a “need for care”. World Psychiatry.

